# Supplementary material for: One-Pot Process: Microwave-Assisted Keratin Extraction and Direct Electrospinning to Obtain Keratin-Based Bioplastic
Source: Int J Mol Sci. 2021 Sep 4;22(17):9597. doi: 10.3390/ijms22179597 (PMC8431758; doi:10.3390/ijms22179597)
Supplement: Supplementary file 1 [file ijms-22-09597-s001.zip › ijms-1298942-supplementary.pdf]

Supplementary Materials

One-pot process: microwave-assisted keratin extraction and direct electrospinning to obtain keratin-based bioplastic

Elena Pulidori [1], Simone Micalizzi [2], Emilia Bramanti [3], Luca Bernazzani [1], Celia Duce [1,*], Carmelo De Maria [2,*], Francesca Montemurro [2], Chiara Pelosi [1], Aurora De Acutis [2], Giovanni Vozzi [2], Maria Rosaria Tinè [1]

1 Department of Chemistry and Industrial Chemistry, University of Pisa, Via G. Moruzzi 13, 56124 Pisa, Italy; elena.pulidori@unipi.it, luca.bernazzani@unipi.it, chiara.pelosi92@gmail.com, mariarosaria.tine@unipi.it

2 Research Center E. Piaggio and Department of Information Engineering, University of Pisa, Largo L. Lazzarino 1, 56126 Pisa, Italy; simone.micalizzi@phd.unipi.it, Francesca.montemurro@unipi.it, a.deacutis@studenti.unipi.it, giovanni.vozzi@unipi.it

3 Institute of Chemistry of Organometallic Compounds, National Research Council, via G. Moruzzi 1, 56124 Pisa, Italy; emilia.bramanti@pi.iccom.cnr.it

* Correspondence: C.D. celia.duce@unipi.it; Tel.: 050 2219311; C.D.M carmelo.demaria@unipi.it; Tel. 050 2217073

TABLE OF THE CONTENTS:

1. **CHARACTERIZATION OF KERATIN EXTRACT**

    1.1. Evaluation of amino acids content (Table S1)

    1.2. SDS-PAGE of TSK fractions (Figure S1)

2. **CHARACTERIZATION OF KERATIN-BASED MATERIAL**

    2.1.Uniaxial tensile test (Figure S2 and S3)

    2.2.Water permeability (Figure S4 and S5)

    2.3. SEM images (Figure S6)

    2.4. TG curves (Figure S7)

    2.5. DSC curves (Figure S8)

    2.6. ATR-FTIR spectra (Figure S9)

1. CHARACTERIZATION OF KERATIN EXTRACT

1.1. Evaluation of amino acids content:

Table S1. Amino acids identify and quantify in the keratin extracts.

	**mg in the keratin extract**							
**Amino acids**	**2h MAE**	**2h MAE**	**5h MAE**	**5h MAE**	**2h CH**	**2h CH**	**5h CH**	**5h CH**
Ser*	/	/	/	/	/	/	/	/
OH-pro*	/	/	/	/	/	/	/	/
Pro	84.62	115.13	104.77	132.40	147.51	161.16	207.23	203.64
Val	51.50	46.17	80.97	58.60	37.73	53.27	71.03	84.35
Met	<LOD**	<LOD	<LOD	<LOD	<LOD	<LOD	<LOD	<LOD
Tyr	0.15	0.16	0.31	0.33	0.21	0.18	0.41	0.42
Leu	<LOD	<LOD	<LOD	<LOD	<LOD	<LOD	<LOD	<LOD
Phe	<LOD	<LOD	<LOD	<LOD	<LOD	<LOD	<LOD	<LOD
Sum	136.27	161.46	186.05	191.33	185.45	214.61	278.68	288.41
Extraction volume (mL)	200	200	200	200	250	250	250	250
**mg/mL**	**0.68**	**0.81**	**0.93**	**0.96**	**0.74**	**0.46**	**1.11**	**1.15**

*Serine and hydroxyproline are not detectable because they coelute with acetic acid.

**LOD: Limit of detection of Pro= 0.069, Val= 0.069, Met= 0.075, Tyr=0.004, Leu=0.774, Phe= 0.020 mg/mL.

1.2. SDS-PAGE of TSK fractions:

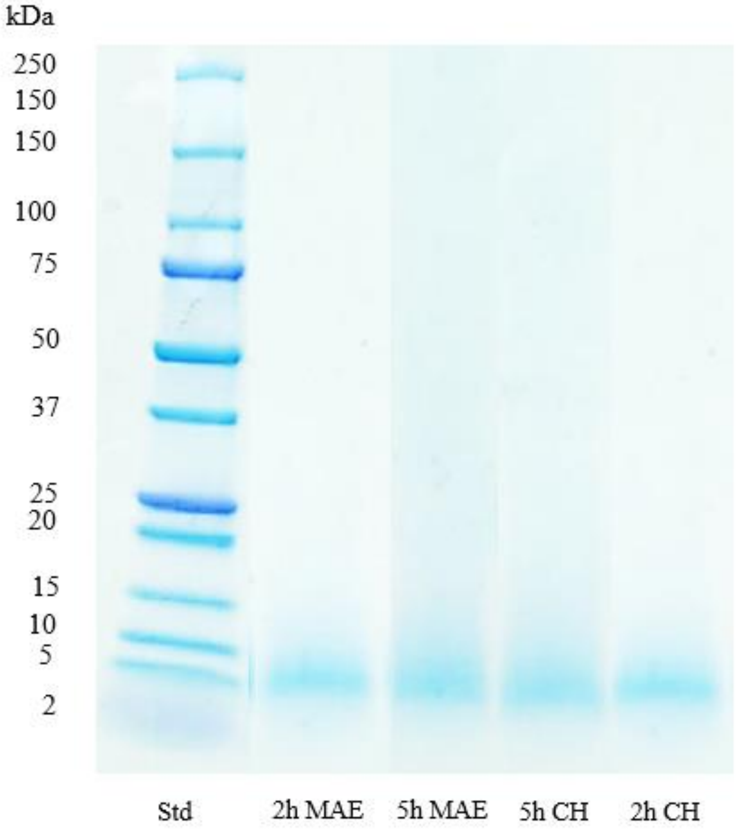

Figure S1. SDS-PAGE of standard proteins (Std) and TSK obtained after 2 and 5 h extraction by MAE and CH process.

2. CHARACTERIZATION OF KERATIN-BASED MATERIAL

2.1. Uniaxial tensile test

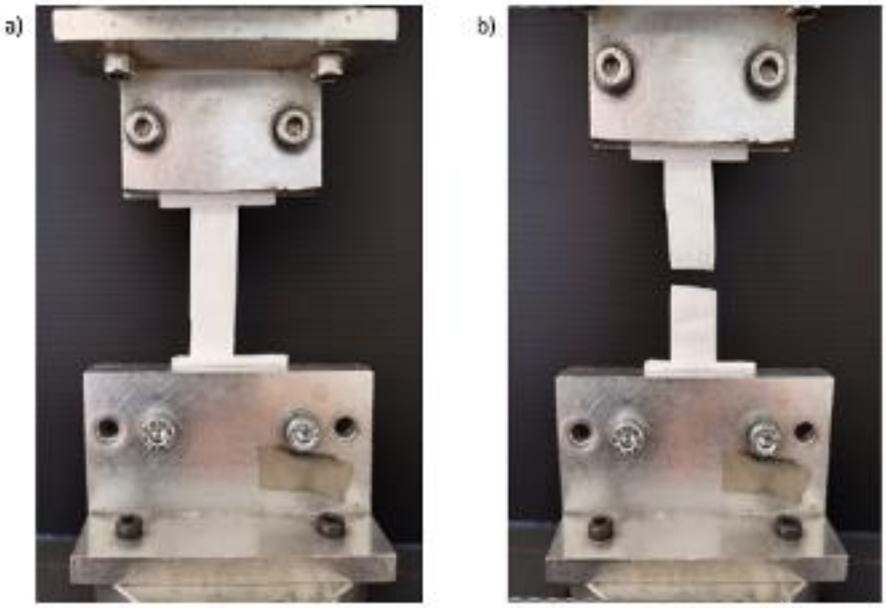

Figure S2. Uniaxial tensile test setup for a keratin-based bioplastic specimen before (a) and after (b) failure.

Figure Description:

Setup for the tensile test of the electrospun keratin-based bioplastics. The specimen is positioned between the two clamps of the machine maintaining a length-to-width ratio of 4:1. The sample is deformed setting a strain rate of 10%/min of the initial length until failure. Notably, the only when the fracture is in the central zone of the specimen (i.e., far from the clams), the test was considered valid.

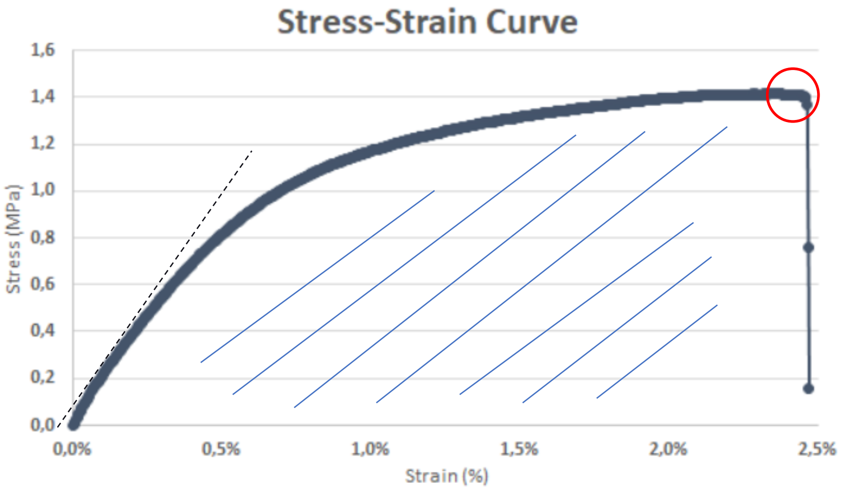

Figure S3. Typical keratin-based bioplastic stress-strain trend resulting during a uniaxial tensile test

Figure description:

The failure stress and strain correspond to ordinate and abscissa values at failure (red circle in figure S4). The elastic modulus is obtained from the slope of the first linear trend and the toughness correspond to area under the curve.

2.2. Water permeability

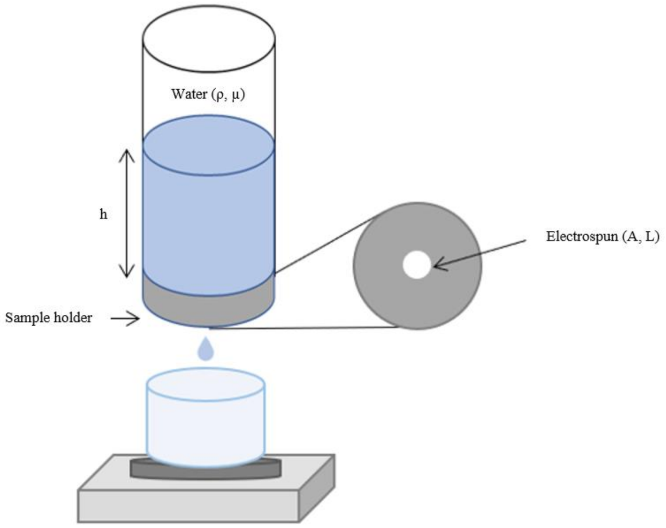

Figure S4. Scheme of the instrument used to evaluate the water permeability.

Figure description:

This device was composed by a vertical tube with a sample holder on its bottom. The electrospun bioplastic sample was placed into the sample holder and the vertical tube was filled with a known amount of water. This operation allowed constant pressure to be applied and driving water through the sample to calculate the permeation time.

The Darcy's law (1) was used to determine the permeability Darcy coefficient:

$$K = \frac{Q\mu L}{A\Delta P} \qquad (1)$$

where, K is the Darcy coefficient [m^2], ΔP [Pa] is the hydrostatic pressure across the membrane, μ is the water viscosity (0.001 Pa·s, 20°C), A is the electrospun surface [m^2], L is the electrospun thickness [m]. The volumetric flow rate Q [m^3/s] was calculated with the follow equation:

$$Q = \frac{Slope}{\rho} \qquad (2)$$

where, the slope is derived from the curve obtained reporting the weight of the water passing through the membrane at four different timepoints (3, 5, 7, and 9 minutes) versus time, and ϱ is the water density (1000 kg/m^3, 20°C). Experiments were performed in triplicate.

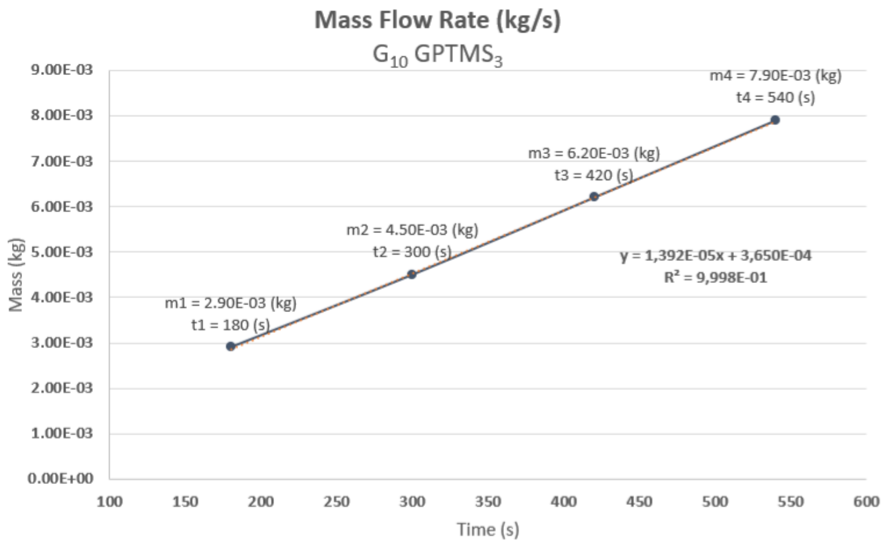

Figure S5. Typical curve obtained during water permeability test, weighting the water passed through the keratin-based bioplastic at 4 time point (180, 300, 420 and 540 s)

Figure description:

Representation of the mass flow rate $\dot{m}$ [kg/s] evaluated as the slope of the curve obtained, during the permeability test, by collecting the weight of the water passing through the membrane at four different timepoints (3, 5, 7, and 9 minutes) versus time. Performing a linear fitting the slope is calculated and then divided by the water density to obtain the volumetric flow rate Q [m^3/s] for the Darcy coefficient calculation.

2.3. SEM images

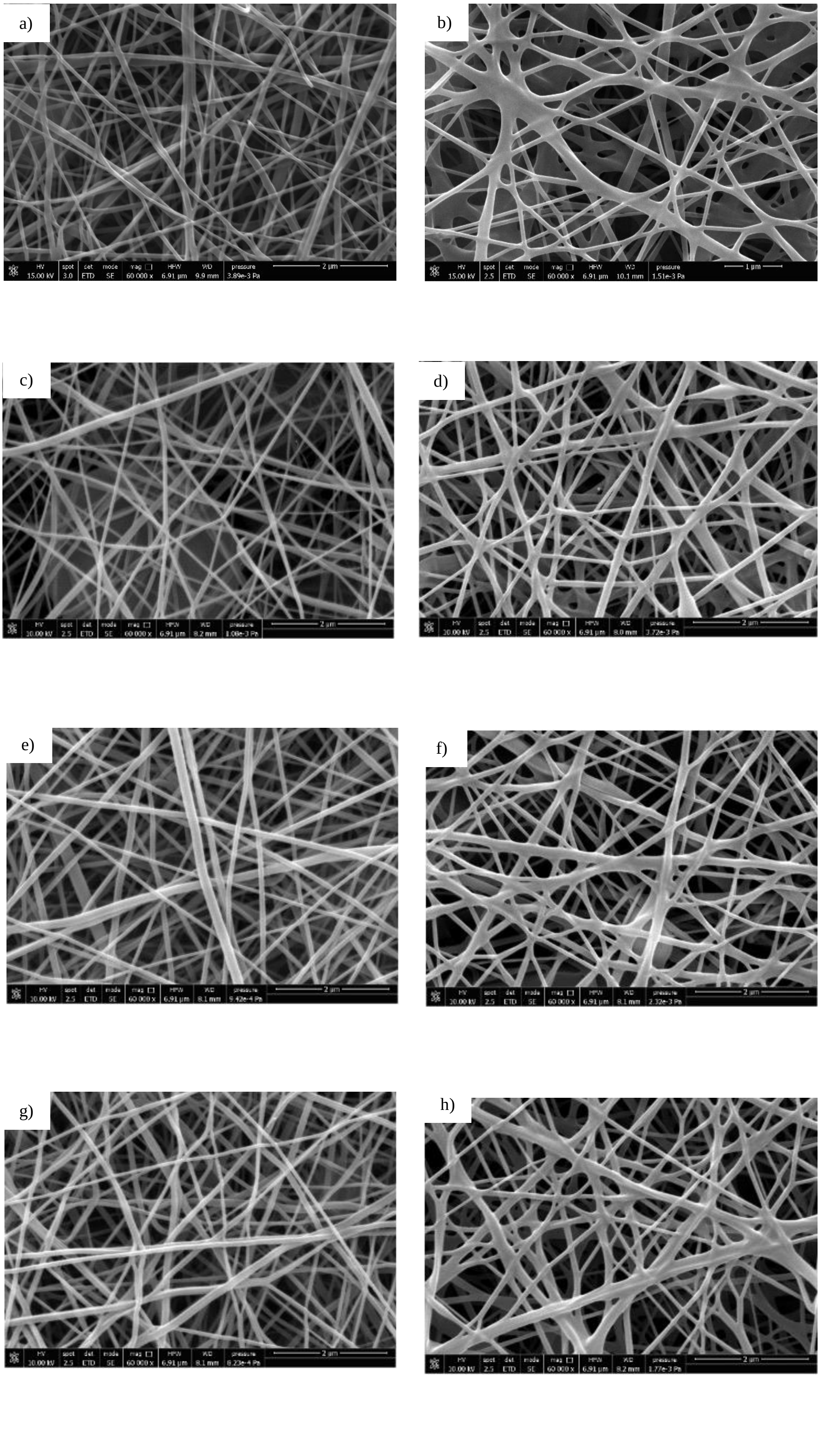

Figure S6. SEM images of $G_{10}GPTMS_3$ (a), $G_{10}GPTMS_6$ (b), $G_{10}K_{0.08}$(2hCH)$GPTMS_3$ (c), $G_{10}K_{0.08}$(2hCH)$GPTMS_6$ (d), $G_{10}K_{0.16}$(5hMAE)$GPTMS_3$ (e), $G_{10}K_{0.16}$(5hMAE)$GPTMS_6$ (f) $G_{10}K_{0.16}$(5hCH)$GPTMS_3$ (g), $G_{10}K_{0.16}$(5hCH) $GPTMS_6$ (h) (magnification 60000x).

2.4. TG curves

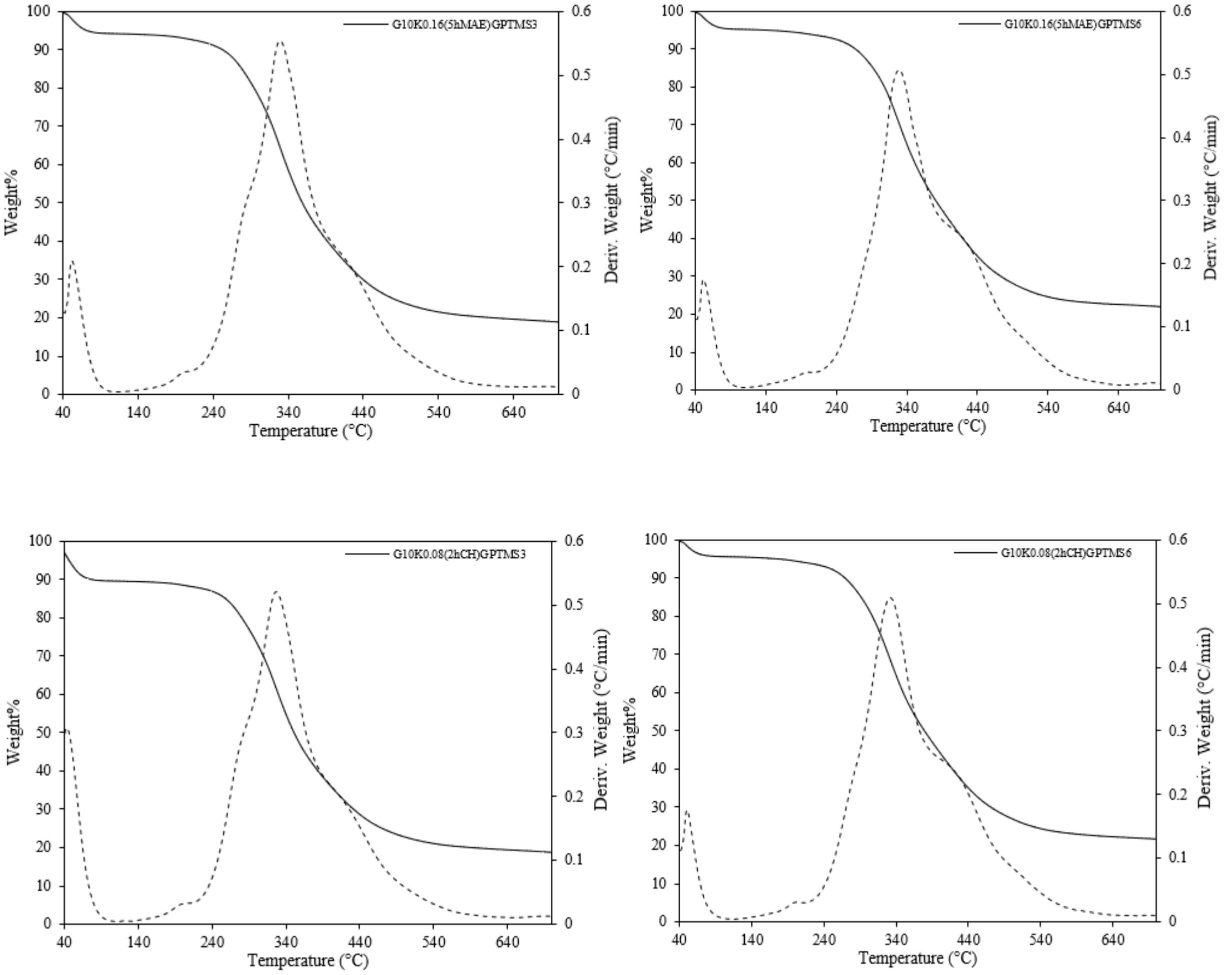

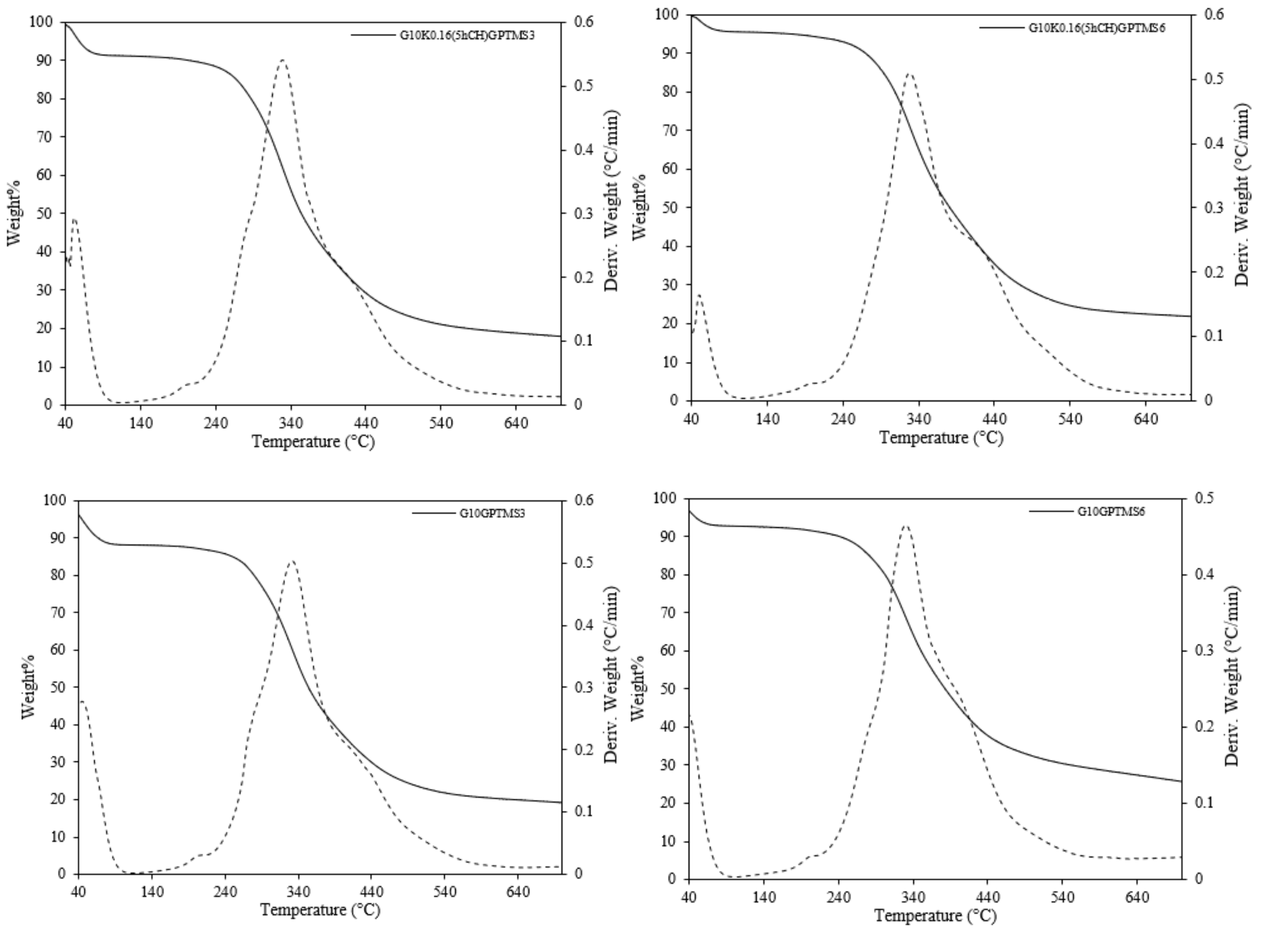

Figure S7. Thermogravimetric curve (solid line) and its DTG (dashed line) under nitrogen flow at 10 °C min^{-1} heating rate of $G_{10}K_{0.16}$(5hMAE)$GPTMS_3$, $G_{10}K_{0.16}$(5hMAE)$GPTMS_6$, $G_{10}K_{0.08}$(2hCH)$GPTMS_3$, $G_{10}K_{0.08}$(2hCH)$GPTMS_6$, $G_{10}K_{0.16}$(5hCH)$GPTMS_3$, $G_{10}K_{0.16}$(5hCH)$GPTMS_6$, $G_{10}GPTMS_3$ and $G_{10}GPTMS_6$ materials.

2.5. DSC curves

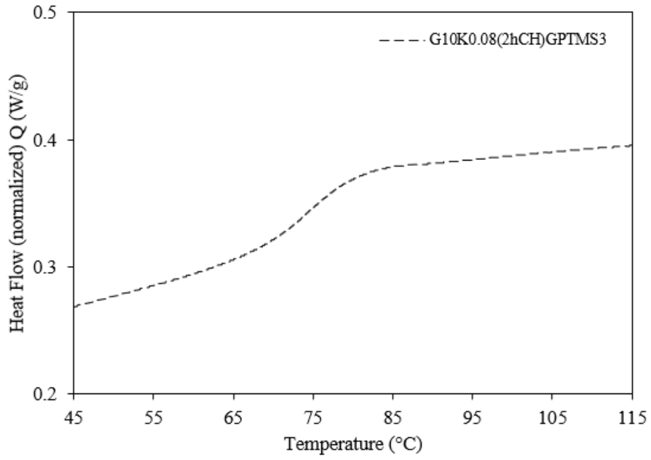

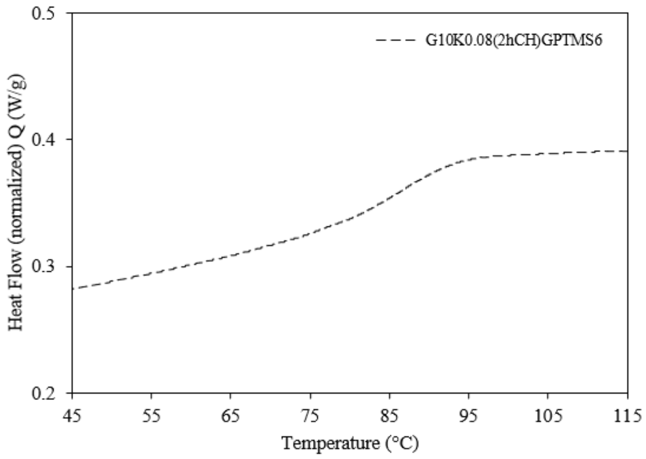

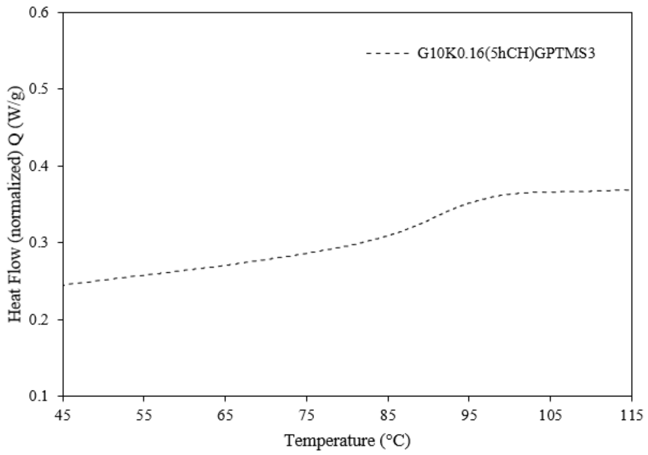

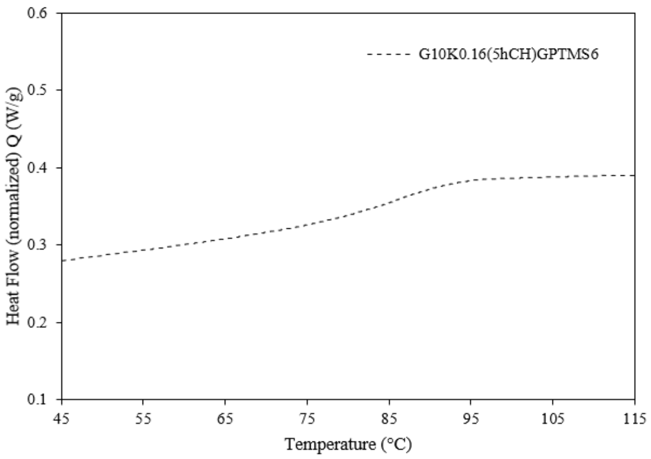

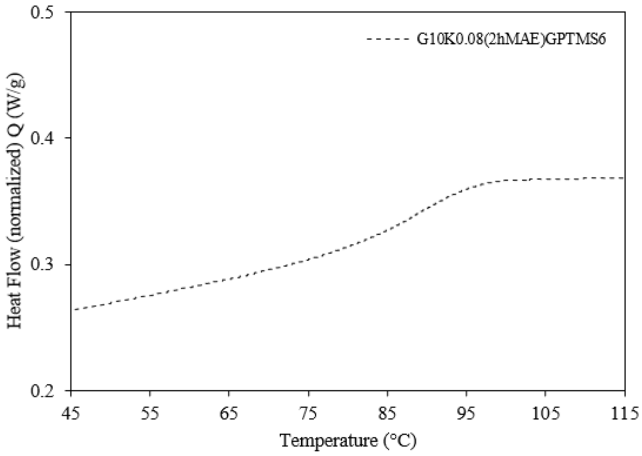

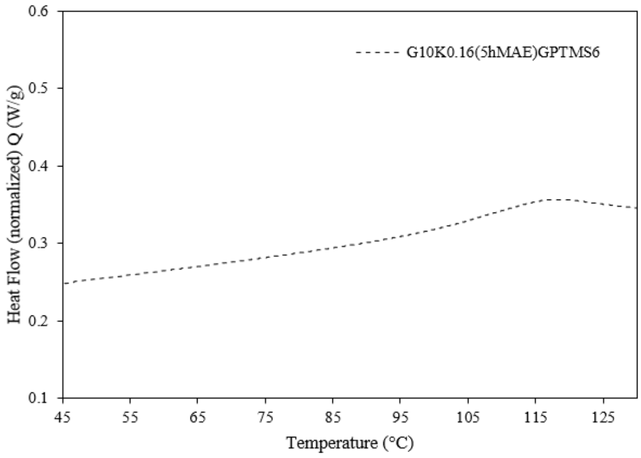

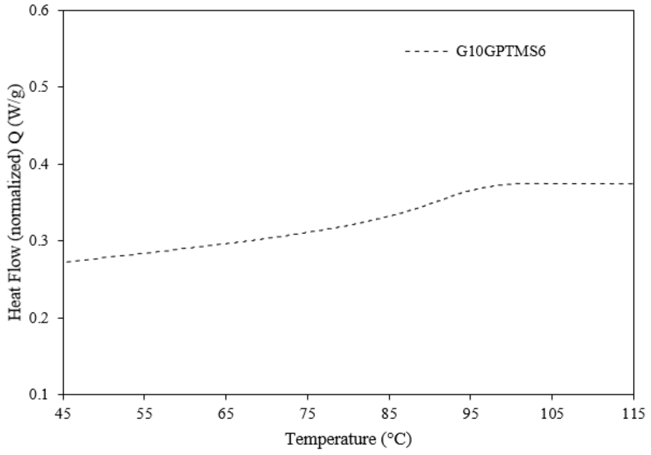

Figure S8. Experimental DSC curves. The heat flow normalized for sample weight obtained in the second heating scan *vs* temperature (heating rate 10°C·min^{-1}) of samples $G_{10}K_{0.08}$(2hCH)$GPTMS_3$, $G_{10}K_{0.08}$(2hCH)$GPTMS_6$, $G_{10}K_{0.16}$(5hCH)$GPTMS_3$, $G_{10}K_{0.16}$(5hCH)$GPTMS_6$, $G_{10}K_{0.08}$(2hMAE)$GPTMS_6$, $G_{10}K_{0.08}$(5hMAE)$GPTMS_6$, $G_{10}GPTMS_6$ is reported.

2.6. ATR-FTIR spectra

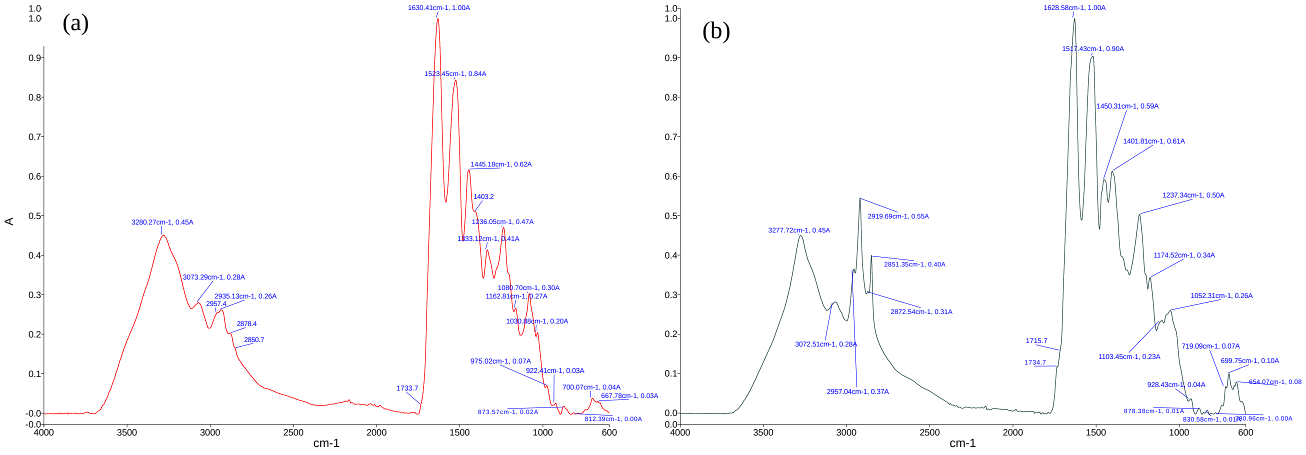

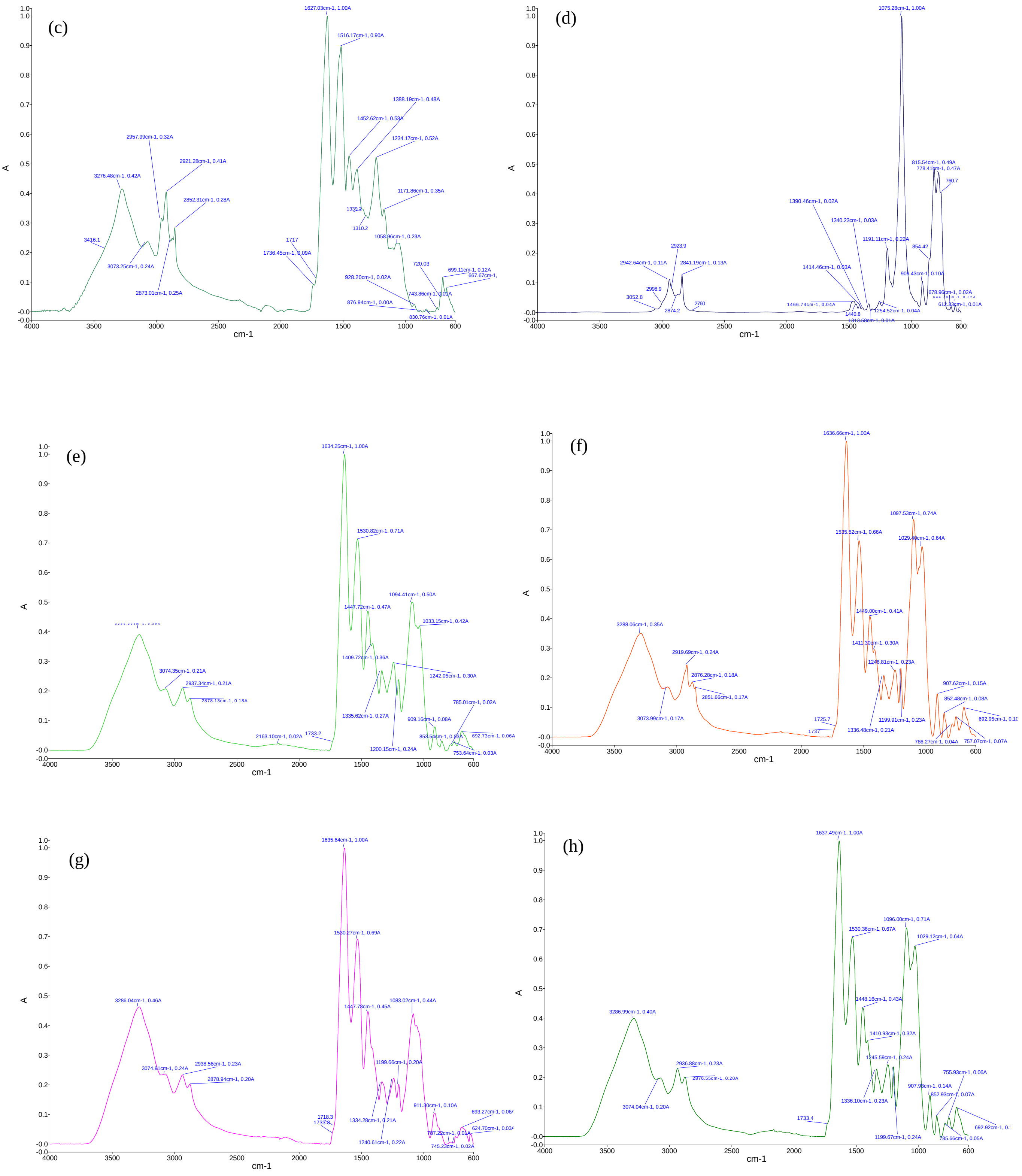

(c)
1627.03cm-1, 1.00A
1516.17cm-1, 0.90A
1388.19cm-1, 0.48A
1452.62cm-1, 0.53A
1234.17cm-1, 0.52A
2957.99cm-1, 0.32A
2921.28cm-1, 0.41A
3276.48cm-1, 0.42A
2852.31cm-1, 0.28A
1171.86cm-1, 0.35A
1339.2
1310.2
3416.1
1058.96cm-1, 0.23A
1717
1736.45cm-1, 0.09A
3073.25cm-1, 0.24A
720.03
699.11cm-1, 0.12A
667.67cm-1,
928.20cm-1, 0.02A
2873.01cm-1, 0.25A
743.86cm-1, 0.01A
876.94cm-1, 0.00A
830.76cm-1, 0.01A
(d)
1075.28cm-1, 1.00A
815.54cm-1, 0.49A
778.41cm-1, 0.47A
760.7
1390.46cm-1, 0.02A
1340.23cm-1, 0.03A
1191.11cm-1, 0.22A
854.42
2923.9
2942.64cm-1, 0.11A
2841.19cm-1, 0.13A
1414.46cm-1, 0.03A
909.43cm-1, 0.10A
2998.9
678.96cm-1, 0.02A
3052.8
2760
1466.74cm-1, 0.04A
612.33cm-1, 0.01A
2874.2
1254.52cm-1, 0.04A
1440.8
1313.58cm-1, 0.01A
(e)
1634.25cm-1, 1.00A
1530.82cm-1, 0.71A
1094.41cm-1, 0.50A
1447.72cm-1, 0.47A
1033.15cm-1, 0.42A
3074.35cm-1, 0.21A
1409.72cm-1, 0.36A
2937.34cm-1, 0.21A
1242.05cm-1, 0.30A
2878.13cm-1, 0.18A
785.01cm-1, 0.02A
1335.62cm-1, 0.27A
909.16cm-1, 0.08A
2163.10cm-1, 0.02A
1733.2
853.54cm-1, 0.03A
692.73cm-1, 0.06A
1200.15cm-1, 0.24A
753.64cm-1, 0.03A
(f)
1636.66cm-1, 1.00A
1097.53cm-1, 0.74A
1535.52cm-1, 0.66A
1029.40cm-1, 0.64A
1449.00cm-1, 0.41A
3288.06cm-1, 0.35A
1411.30cm-1, 0.30A
2919.69cm-1, 0.24A
1246.81cm-1, 0.23A
2876.28cm-1, 0.18A
907.62cm-1, 0.15A
2851.66cm-1, 0.17A
852.48cm-1, 0.08A
3073.99cm-1, 0.17A
1199.91cm-1, 0.23A
1725.7
1737
1336.48cm-1, 0.21A
786.27cm-1, 0.04A
757.07cm-1, 0.07A
(g)
1635.64cm-1, 1.00A
1530.27cm-1, 0.69A
3286.04cm-1, 0.46A
1447.78cm-1, 0.45A
1083.02cm-1, 0.44A
1199.66cm-1, 0.20A
3074.91cm-1, 0.24A
2938.56cm-1, 0.23A
2878.94cm-1, 0.20A
911.30cm-1, 0.10A
1718.3
1733.8
1334.28cm-1, 0.21A
787.22cm-1, 0.01A
1240.61cm-1, 0.22A
745.23cm-1, 0.02A
624.70cm-1, 0.03A
(h)
1637.49cm-1, 1.00A
1096.00cm-1, 0.71A
1530.36cm-1, 0.67A
1029.12cm-1, 0.64A
1448.16cm-1, 0.43A
3286.99cm-1, 0.40A
1410.93cm-1, 0.32A
1245.59cm-1, 0.24A
2936.88cm-1, 0.23A
2876.55cm-1, 0.20A
755.93cm-1, 0.06A
907.93cm-1, 0.14A
852.93cm-1, 0.07A
3074.04cm-1, 0.20A
1336.10cm-1, 0.23A
1733.4
1199.67cm-1, 0.24A
785.66cm-1, 0.05A
A
cm-1

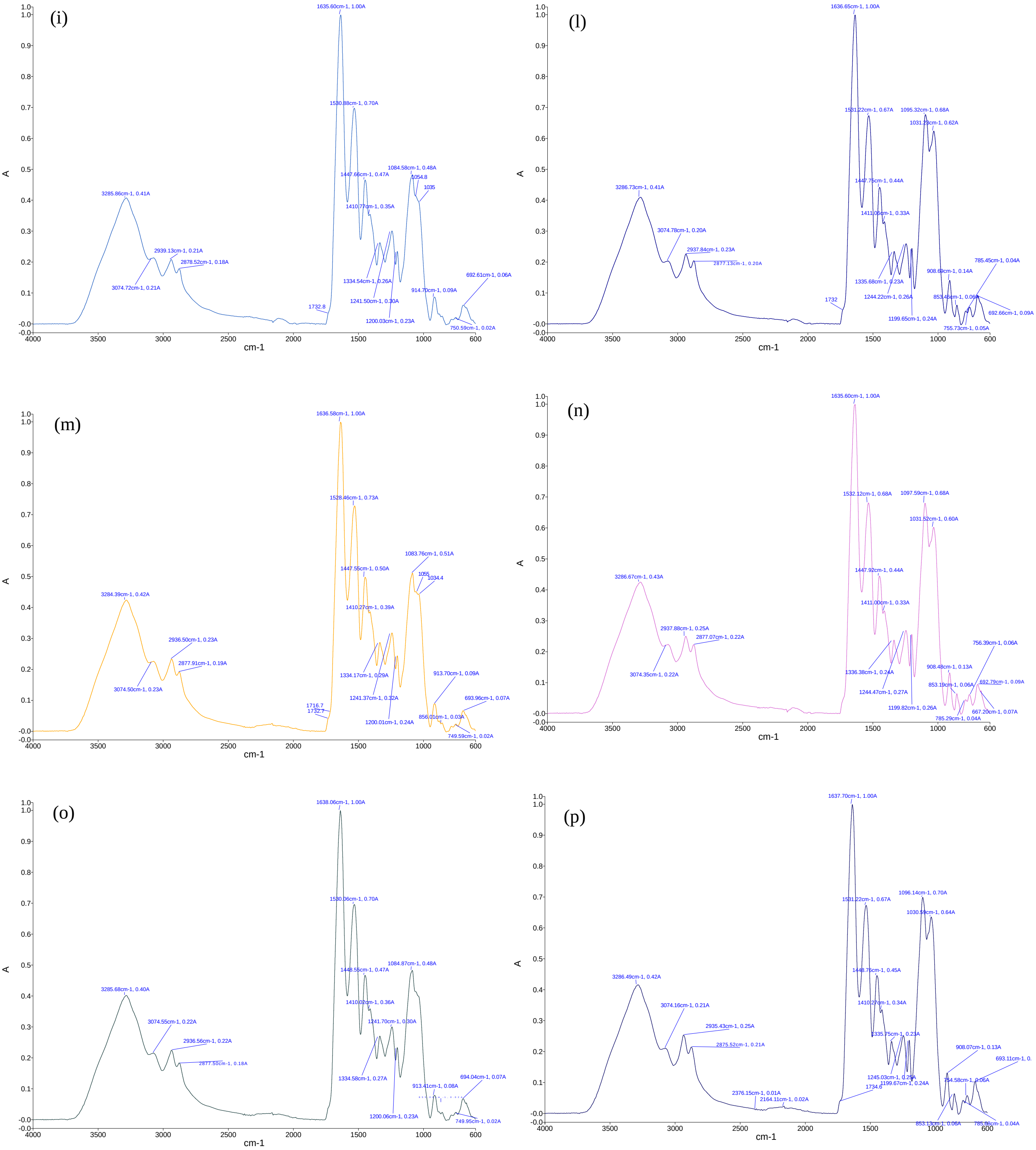

Figure S9. ATR-FTIR spectra of gelatine powder (a), SK_{IP} obtained from 2h MAE extract (b) and 5h MAE (c), GPTMS (d), $G_{10}GPTMS_3$ (e), $G_{10}GPTMS_6$ (f), $G_{10}K_{0.08}(2hMAE)GPTMS_3$ (g),

$G_{10}K_{0.08}$(2hMAE)$GPTMS_6$ (h), $G_{10}K_{0.16}$(5hMAE)$GPTMS_3$ (i), $G_{10}K_{0.16}$(5hMAE)$GPTMS_6$ (l), $G_{10}K_{0.08}$(2hCH)$GPTMS_3$ (m), $G_{10}K_{0.08}$(2hCH)$GPTMS_6$ (n), $G_{10}K_{0.16}$(5hCH)$GPTMS_3$ (o), $G_{10}K_{0.16}$(5hCH)$GPTMS_6$ (p) materials in the 4000-600 cm^{-1} regions.
